# Encapsulating peritoneal sclerosis post liver transplant and peritoneal dialysis: case report and literature review

**DOI:** 10.1093/jscr/rjad193

**Published:** 2023-04-18

**Authors:** Rodrigo Piltcher-da-Silva, João Francisco Petry, Ana Laura de Araujo Freitas, Guilherme Vieceli Rhoden, Gabriel Jasinski, Mariana Piltcher-Recuero, Luiz Francisco Cravo Bettini, Yan Sacha Hass Aguilera, Juliana El Ghoz Leme, Marco Aurélio Raeder da Costa, Júlio Cezar Uili Coelho

**Affiliations:** General and Digestive Surgery Service, Hospital Nossa Senhora das Graças, Curitiba, PR, Brazil; General and Digestive Surgery Service, Hospital Nossa Senhora das Graças, Curitiba, PR, Brazil; General and Digestive Surgery Service, Hospital Nossa Senhora das Graças, Curitiba, PR, Brazil; General and Digestive Surgery Service, Hospital Nossa Senhora das Graças, Curitiba, PR, Brazil; General and Digestive Surgery Service, Hospital Nossa Senhora das Graças, Curitiba, PR, Brazil; General and Digestive Surgery Service, Hospital Nossa Senhora das Graças, Curitiba, PR, Brazil; General and Digestive Surgery Service, Hospital Nossa Senhora das Graças, Curitiba, PR, Brazil; General and Digestive Surgery Service, Hospital Nossa Senhora das Graças, Curitiba, PR, Brazil; Nefrology Service, Hospital Nossa Senhora das Graças, Curitiba, PR, Brazil; General and Digestive Surgery Service, Hospital Nossa Senhora das Graças, Curitiba, PR, Brazil; General and Digestive Surgery Service, Hospital Nossa Senhora das Graças, Curitiba, PR, Brazil

## Abstract

Encapsulating peritoneal sclerosis (EPS) is a rare and debilitating condition. A fibrocollagenous membrane, which promotes encasement of the small intestine leaving a cocoon-like appearance, takes place. It is mainly associated with peritoneal infections, medications, peritoneal dialysis and systemic inflammatory diseases. Diagnosis is based on clinical history, intestinal obstruction and imaging exam. We report a case of EPS in a 68-year-old man with a medical history of liver transplantation and peritoneal dialysis, complaining of obstructive bowel symptoms.

## INTRODUCTION

Encapsulating peritoneal sclerosis (EPS) is a condition in which a fibrocollagenous membrane promotes the encasement of the bowel as a consequence of multiple inflammatory processes [1, 2]. It is a rare cause of intestinal obstruction with an unknown incidence. It occurs in 0.7–13.6/1000 patients-years in peritoneal dialysis (PD) patients [3]. There is equatorial predilection and men are more affected than women in a 2:1 ratio [4].

The pathogenesis is not well understood; however, it is related to fibrogenesis and increased endothelial permeability, which will promote exuberant fibrin deposition on the peritoneum [2, 5]. EPS is commonly associated with PD or multiple peritoneal insults [1, 4]. Nonetheless, genetic predisposition explains why few patients with recurrent peritoneal insults develop EPS and why some develop after a single peritoneal event [1]. The risk factors are exposed in Table 1 [1, 2, 4].

**Table 1:** Risk factors

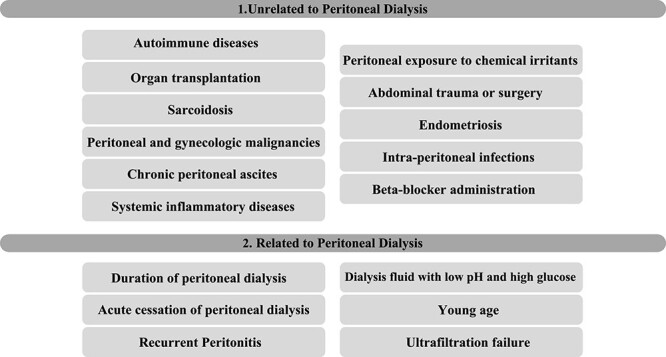

## PRESENTATION OF CASE

A 68-year-old man was seen in the emergency department complaining of 1 day of abdominal pain and distension, change in bowel habits, nausea and vomiting. He underwent liver transplantation for cirrhosis due to autoimmune hepatitis in 2007, and has been treated for systemic hypertension, epilepsy, portal vein thrombosis and chronic kidney disease, managed with PD for 8 months. There is no history of variceal bleeding nor spontaneous bacterial peritonitis. He was in use of mycophenolate, oxcarbazepine, spironolactone, pantoprazole and propranolol.

**Figure 1:**
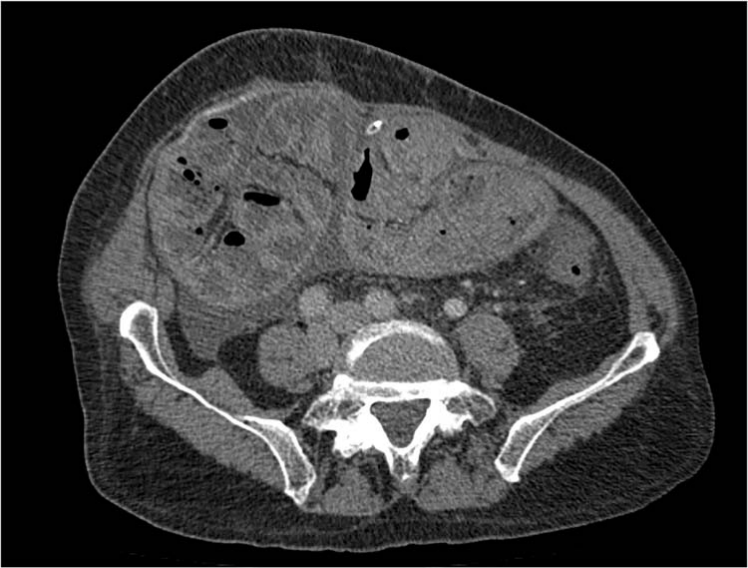
Portal phase transversal section image on CT showing loops wrapped in a membrane-like structure and thickened peritoneum.

**Figure 2:**
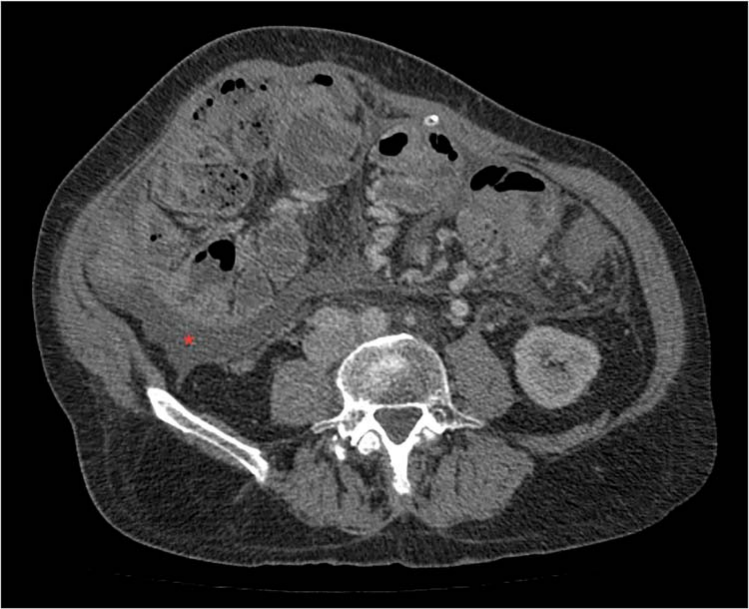
Portal phase transversal section image on CT showing membrane-like structure and loculated ascites (asterisk).

**Figure 3:**
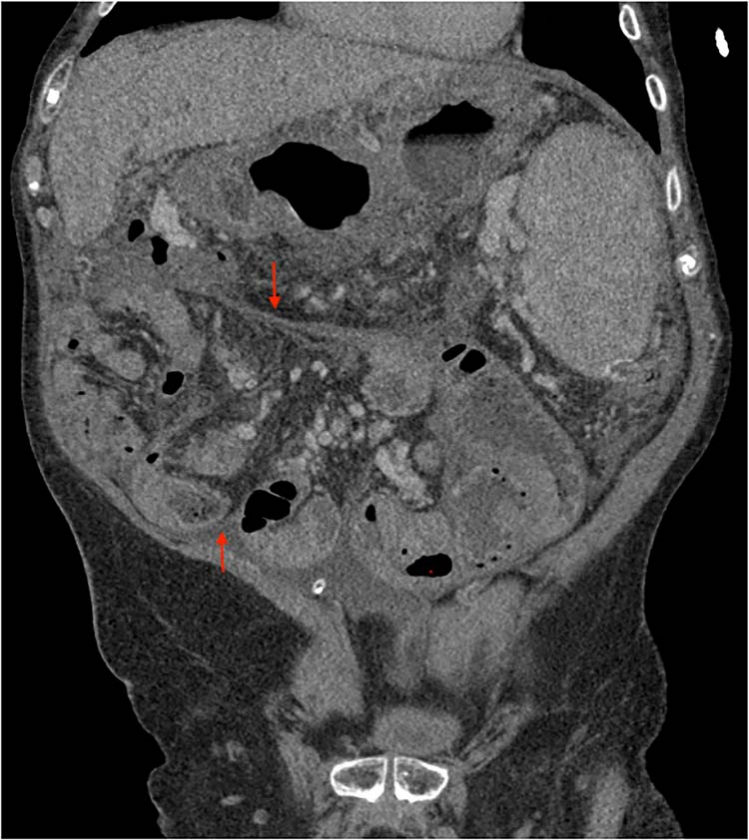
Portal phase coronal section image on CT showing loops wrapped in a membrane-like structure (arrow).

His clinical history and contrast-enhanced computed tomography (CT) findings (Figs 1–3) were compatible with EPS in Stage 4 (Table 2). Laboratory tests were non-specific, showing inflammation and malnutrition. He underwent nutritional support, corticosteroids and surgery. In surgery, fibrocollagenous membrane resection and enterolysis were done. Postoperative period was complicated by pneumonia treated with piperacillin+tazobactam and later bacterial peritonitis treated with ertapenem. The patient was discharged after 29 days with adequate nutrition and asymptomatic.

**Table 2:** Stages of encapsulating peritoneal sclerosis (EPS)

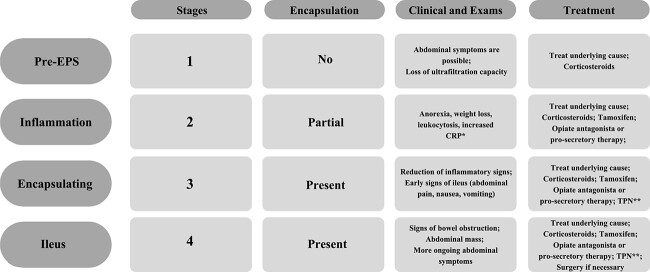

## DISCUSSION

EPS was first described in 1868 by Cleland [4, 6] and named in 1907 by Owtschinnikow as peritonitis chronica fibrosa incapsulata [4, 7]. The trigger for this syndrome is the repetitive peritoneal insults and fibrocollagenous membrane formation [2]. However, only genetic predisposition explains why only few patients with recurrent peritoneal insults develop EPS and why some patients develop after a single inflammatory event [1].

The process precipitates a proinflammatory cytokines (IL-1, IL-6, IL-18) cascade with activation of transforming growth factor β1, platelet-derived growth factor and vascular endothelial growth factors [2, 8]. Transdifferentiation of peritoneal mesothelial to mesenchymal cells and increased extracellular matrix components (collagen type 1) is the result [2]. Mesothelial cell layer with fibroblast proliferation, fibrocollagenous and fibrin deposition results in fibrocollagenous membrane. Mononuclear cell infiltration is present if there is active inflammation [1, 2, 9]. This whole process is strongly associated with PD for prolonged time, with rare occurrence before 3 years (1–3). In our case, it occurred in 8 months after PD was initiated; however, he has hepatic transplantation as a concomitant risk factor (Table 1).

EPS presents with a variety of unspecific symptoms, such as nausea and vomiting, abdominal pain, abdominal distention and weight loss [2–3]. The onset of symptoms is late, making malnutrition a common finding (75%) [2, 10]. Laboratory is non-specific, showing alteration related to infection, malnutrition and inflammation [11]. Histopathological findings have overlap with simple peritoneal sclerosis or peritonitis [3].

Imaging exams, mainly CT are important to evaluate causes of bowel obstruction [4]. Dilated or non-dilated small intestine loops may be wrapped in a membrane-like structure, proximal bowel dilatation, thickened peritoneum with diffuse or local calcification, and loculated ascites are some of the findings on CT [1, 12]. Barium X-ray provides a clue to bowel encapsulation, showing clustered loops of the small intestine in the center of the abdomen, known as the cauliflower sign [4]. Ultrasound and magnetic resonance imaging may give a clue but are rarely used [1, 12].

EPS is classified based on symptoms, inflammation, membrane-like formation and CT findings into four sequential stages (Table 2). The treatment has been guided based on this classification [2, 13].

The beginning of treatment is to stop peritoneal injury, like PD cessation, and nutritional support [1]. Immunosuppression is the next step. Corticosteroids are the best studied and recommended for those with active inflammation, based on clinical observation and laboratory exams [14]. Corticosteroids are stopped if there is no improvement after 1 month of treatment. Other immunosuppressive medications could be used, such as everolimus which also has an antiproliferative effect [15].

Tamoxifen, a drug with anti-fibrotic properties is recommended on Stage 3; however, it is based on observational studies. One retrospective study reported a significant difference in mortality (45.8 vs. 74.4%, *P* < 0.05) after 130 months [16] and others found no benefit [17]. Surgery is needed in advanced stages to treat bowel obstruction and malabsorption, with resection of the fibrocollagenous membrane, enterolysis and enterectomy if needed. The post-surgical mortality is up to 35.4% [1, 2, 18].

## CONCLUSION

EPS is a rare and progressive syndrome, its recognition is based on high clinical suspicion, careful history and CT findings. The sooner the treatment begins, the better the patient’s general condition is expected to improve and lower the mortality rate. Further research is needed to improve our knowledge about this syndrome and its management.
